# Transporter function and cyclic AMP turnover in normal colonic mucosa from patients with and without colorectal neoplasia

**DOI:** 10.1186/1471-230X-12-78

**Published:** 2012-06-26

**Authors:** Karen Kleberg, Gerda Majgaard Jensen, Dan Ploug Christensen, Morten Lundh, Lars Groth Grunnet, Svend Knuhtsen, Steen Seier Poulsen, Mark Berner Hansen, Niels Bindslev

**Affiliations:** 1 Department of Biomedical Sciences, Faculty of Health and Medical Sciences, University of Copenhagen, Copenhagen, Denmark; 2 Department of Surgery K, Bispebjerg Hospital, University of Copenhagen, Copenhagen, Denmark

**Keywords:** Cyclic-AMP compartmentalization, Human colonic biopsy, OATP-, ABC-, PGE_2_-transporters

## Abstract

**Background:**

The pathogenesis of colorectal neoplasia is still unresolved but has been associated with alterations in epithelial clearance of xenobiotics and metabolic waste products. The aim of this study was to functionally characterize the transport of cyclic nucleotides in colonic biopsies from patients with and without colorectal neoplasia.

**Methods:**

Cyclic nucleotides were used as model substrates shared by some OATP- and ABC-transporters, which in part are responsible for clearance of metabolites and xenobiotics from the colonic epithelium. On colonic biopsies from patients with and without colorectal neoplasia, molecular transport was electrophysiologically registered in Ussing-chamber set-ups, mRNA level of selected transporters was quantified by rt-PCR, and subcellular location of transporters was determined by immunohistochemistry.

**Results:**

Of four cyclic nucleotides, dibuturyl-cAMP induced the largest short circuit current in both patient groups. The induced short circuit current was significantly lower in neoplasia-patients (p = 0.024). The observed altered transport of dibuturyl-cAMP in neoplasia-patients could not be directly translated to an observed increased mRNA expression of OATP4A1 and OATP2B1 in neoplasia patients. All other examined transporters were expressed to similar extents in both patient groups.

**Conclusions:**

OATP1C1, OATP4A1, OATP4C1 seem to be involved in the excretory system of human colon. ABCC4 is likely to be involved from an endoplasmic-Golgi complex and basolateral location in goblet cells. ABCC5 might be directly involved in the turnover of intracellular cAMP at the basolateral membrane of columnar epithelial cells, while OATP2B1 is indirectly related to the excretory system. Colorectal neoplasia is associated with lower transport or sensitivity to cyclic nucleotides and increased expression of OATP2B1 and OATP4A1 transporters, known to transport PGE_2_.

## Background

Colorectal neoplasia (CRN) and colorectal cancer (CRC) still pose major challenges concerning etiology, early diagnosis, prognosis, and treatment. Deaths due to colorectal cancer rank among the highest cancer related mortalities [1]. Recently, epithelial transporter dysfunctions of the colon have attracted attention in relation to development of CRN and CRC [2-4]. The colon has a molecular excretory system build to excrete xenobiotics, toxins and waste metabolites in parallel with excretory systems found in kidney and liver [4]. Many molecular transporters are conceived to take part in this excretory paradigm of the colon. Several of these transporters are also considered important for controlling the level of intracellular signaling molecules [5,6]. Selective basolateral and apical molecular transporters of normal colonic epithelial cells are furthermore believed to be important in maintenance of a healthy micro-environment, while dysfunction of these excretory mechanisms is suspected to cause a cytotoxic and potentially oncogenic milieu [6]. Special attention has been paid to members of the large ATP-binding cassette family (ABC-transporters) [2]. An association of these transporters to cancer is well established with respect to response to anti-cancer treatment and prognostics [4]. Studies on a possible connection to cancer development by other types of transporters such as the organic anion transporting polypeptides (OATPs) are now being initiated as well [5,7-10]. However, the function and build-up of the colonic excretory system including its transporters are yet insufficiently characterized.

Recently, second messengers in form of cyclic nucleotides (cNTs) such as cAMP and cGMP have turned out as shared substrates for some OATP- and ABC-transporters [11,12]. Both transporter families have members characterized by substrate promiscuity and overlap [13]. The importance of cellular import and export of cNTs is still debated, though gradually accepted as a mean to regulate intracellular levels of these signaling molecules [11,12]. cNT influx is thought to occur by transporter OATP4C1, newly reported to influx cAMP over the basolateral membrane of kidney tubule cells and also present in the human colon [11,14]. Known mediators of cNT-efflux, as ABCC4, ABCC5 and ABCC8, are expressed in colonic tissue together with other ABC-transporters as established by rt-qPCR [15,16]. Meanwhile, it is unknown which transporters are involved in the turnover of cNTs in colon epithelial cells and which colonic transporters are involved in functions possibly related to cancer progression and prognostics [14].

It has become easy to assess transporter function and secretion in *in vitro* human colon epithelium by means of the mini-Ussing air suction (MUAS)-chamber technique [17]. And, since colonic secretion is induced by cAMP, which is likely transported basolaterally to the interior by presence of the OATP4C1-influxer, we have taken advantage of this transfer to study the effect of externally applied cNTs on the induction of colonic secretion and possible excretion.

The overall aim of this study had two objectives. One objective was to functionally characterize the excretory system of human colon and to investigate whether alterations or dysfunctions in this molecular excretory system is associated with CRN and CRC pathogenesis. The second objective was an initial characterization of the turnover processes of second messenger cAMP, related to transporters in human colonic mucosa.

We conclude that transporters as OATP1C1, OATP4A1, OATP4C1 seem to be involved in the excretory system of human colon. ABCC4 is also involved but from an endoplasmic-Golgi complex location, while OATP2B1 is indirectly related to the excretory system. ABCC5 might be directly involved in the turnover of intracellular cAMP. Colonic epithelium from CRN-patients had lower transport or sensitivity to cyclic nucleotides and an increased expression of two PGE_2_-transporters, OATP2B1 and OATP4A1; however it is premature to draw firm conclusions based on this latter observation.

## Methods

### Study population and biopsy procedure

Patients, 50 years or older and referred to colonoscopy, were enrolled in the study. Medical history was recorded according to case notes. Patients with either a history of or present colorectal neoplasia were included in the neoplasia group, whereas patient with no present or former neoplasia were placed in the control group irrespective of other extra-intestinal diagnoses. Patients were excluded from the study in case of history of any kind of chronic inflammatory condition of the intestine or if they had recently been or were presently undergoing a radiation and/or chemotherapy for CRN. A total of 19 patients were enrolled, with 11 subjects in the neoplasia group and 8 subjects in the control group. There were four women in each group and the mean age was 67 ± 13 yrs in the control group and 69 ± 4 yrs in the neoplasia group.

Six biopsies from each patient were obtained during endoscopy from macroscopically normal appearing mucosa using standard biopsy forceps (Radial Jaw 3) from Boston Scientific with an outside diameter of 2.2 mm. The biopsies were obtained approximately 30 cm from the anus and at least 10 cm from macroscopically abnormal tissue on retraction of the endoscope. Biopsies were immediately transferred to an iced, oxygenized bicarbonate Ringer solution with the following composition (in mM): Na^+^ (140), Cl^-^ (117), K^+^ (3.8), PO_4_^-^ (2.0), Mg^2+^ (0.5), Ca^2+^ (1.0) and HCO_3_^-^ (25). pH was adjusted to 7.4 by gassing the media with 95% O_2_/5% CO_2_.

### Ethics

The study protocol was approved by the Scientific Ethical Committee of Copenhagen (KA 97161) and Frederiksberg Counties (KF01-232/03) and conducted in accordance with the Helsinki declaration. All patients participating gave written informed consent.

### Chemicals

Theophylline, indomethacin, acetazolamide, NPPB, SITS, rifamycin SV, cAMP and cGMP were purchased from Sigma-Aldrich (Seelze, Germany). db-cAMP, db-cGMP and OATP antibodies were purchased from Santa Cruz Biotechnology (Santa Cruz, CA, USA), OATP1C1 (cat. no.: sc-51350), OATP2B1 (cat. no.: sc-66561), OATP4A1 (cat. no.: sc-51169), OATP4C1 (cat. no.: sc-136775). Bumetanide was received as a gift from Leo Pharmaceuticals (Copenhagen, Denmark). ABC-antibodies were obtained from LifeSpan Biosciences (Seattle, WA, USA), ABCB1 (cat. no.: LS-A9403-50), ABCC3 (cat. no.: LS-C16477-250), ABCC4 (cat. no.: LS-B4-50), and ABCC5 (cat. no.: LS-B2334-50). Oligo primers were obtained from Tag Copenhagen (Copenhagen, Denmark). Normal goat and rabbit serum was purchased from Dako (Glostrup, Denmark). All other chemicals were of analytical grade.

### Functional characterization in MUAS-chamber

Biopsies were mounted in MUAS-chambers as previously described [17]. Pretreatment of the biopsies was performed 15 min mounting. Amiloride (100 μM, apically) was added to inhibit ENaC-mediated sodium transport. Theophylline (400 μM, apically and basolaterally) was added to dampen endogenous breakdown of cNTs. Indomethacin (40 μM, basolaterally) was added to inhibit endogenous synthesis of cNTs. Experiments were conducted after additional 20 min equilibration. cAMP, cGMP, dibutyryl-cAMP and dibutyryl-cGMP were used as model substrates of OATP and ABC-transporters and applied either luminally or basolaterally (500 μM). Each experiment was terminated by basolateral addition (in μM) of bumetanide (25), NPPB (500), SITS (1000), and acetazolamide (250), alone or in combination. In a few experiments after pretreatment, rifamycin SV (50–100 μM) was added to either side of the tissue to test for its inhibition of cNT influx.[18,19]. DMSO was the vehicle for applying lipid soluble drugs and its final concentration always kept below 1 per mille in chambers. A thorough discussion concerning the validity of measured SCC and slope conductance by the MUAS-method is presented earlier [17].

After completion of the study the biopsies were gently dismounted and preserved in 4% paraformaldehyde for subsequent histological assessment of tissue integrity. Epithelium preservation on biopsies was scored on a scale from 0 to 3, where 0 is overall intact epithelial surface, 1: slightly damaged epithelium, 2: at least 1/3 intact epithelium and 3: practically no intact epithelial surface.

### RNA isolation

From each patient, one biopsy was frozen on dry ice and saved at −80 °C for rt-qPCR studies. For RNA isolation, biopsies were homogenized using a Polytron (PT 1600 E) for 2 min. RNA isolation was performed using a NucleoSpin® RNA/Protein kit from Macherey-Nagel (Düren, Germany). RNA concentration was determined with NanoDrop® ND-1000 from NanoDrop Technologies (Wilmington, DE, USA), and the purity assessed by means of absorbance ratios (A_260_/A_230_ and A_260_/A_280_).

### Primer design

Rt-qPCR for eight transporter genes was performed on biopsies from 6 patients in both groups using β-actin as reference gene. The following eight transporters were selected based on previous reports on presence in human colonic mucosa: OATP1C1, OATP2B1, OATP4A1, OATP4C1, ABCB1, ABCC3, ABCC4, and ABCC5 [14]. Gene sequences were retrieved via http://www.ensemble.org and the primers were designed using Primer 3 software. NetPrimer software was used to identify and exclude primers with a tendency to dimer and/or hairpin formation.

Selected primers were: β-actin (forward: ACCCAGCACAATGAAGATCA, reverse: CGTCATACTCCTGCTTGCTG), OATP1C1 (forward: CCTCAGAAGAAAAGCAACCAT reverse: ACCATCAATAACTCCCACCAG), OATP2B1 (forward: TGATCTGCTTCGCCTTAGTTT, reverse: CTGGATCTGCTCTCTTTGGTC), OATP4A1 (forward: TTCCTGATGACAGAACAGTGC, reverse: ACAGCCGACTTTAAACCACAG), OATP4C1 (forward: GGCTTTCTGCTTCACTACTGC, reverse: CATAACGCTTCTCAACAGTGG), ABCB1 (forward: TGGATTCATCAGCTGCATTT, reverse: TGATGGAGTCATTGTGGAGAA), ABCC3 (forward: TACTCTCTGCCCTCATCTTGG, reverse: AAAACAGGCGGGAGAGAAA), ABCC4 (forward: CCCTCACTGAAACAGCAAAA, reverse: TAGTTAAGGTCGAGGGCTGTC), ABCC5 (forward: GCTCTTCTTGCCACAGTCTCT, reverse: TTTTCGTGGCTTTCTTCTCTG).

### cDNA synthesis

cDNA synthesis was conducted using an iScript™ cDNA Synthesis Kit from Bio-Rad (Copenhagen, Denmark) according to manufactures instructions as previously described [20].

### Rt-qPCR

For rt-qPCR, cDNA was amplified using a Fast SYBR® Green Master Mix (Applied Biosystems). Samples were run in four dilutions (0.05, 0.5, 5.0, and 10% cDNA v/v) on 384 well plates in volumes of 10 μL and primer and master mix concentrations of 0.6 μM and 50%v/v, respectively. All samples were run in triplicate with β-actin on all plates. Amplification was performed using a 7900HT Fast Real-Time PCR System from Applied Biosystems (Foster City, CA, USA) in accordance with the supplier’s manual. Standard and dissociation curves confirmed acceptable amplification efficiencies and specificities of the primer sets. The expression profiles were calculated using the deltaCT-method in which the amplification calculation is adjusted for primer-set efficiencies [21]. Results were analyzed using adjuvant SDS 2.3 software. Results are based on the 10% cDNA dilution.

### Transporter localization

One colon biopsy from each patient was put aside in 4% neutral buffered formaldehyde after the endoscopic procedure. Biopsies were subsequently embedded in paraffin and cut in 10 μm thin slices. Immunohistochemical localization of transporters was performed on biopsies from two patients in each group. The sections were deparaffinated and rehydrated, followed by heat treatment in a microwave oven in order to unmask epitopes. The sections were blocked with a 2% bovine serum albumin solution for 10 minutes, to rule out unspecific antibody adhesion, followed by incubation with a primary antibody at 4 C overnight in the following concentrations: OATP1C1 (1:100), OATP2B1 (1:100), OATP4A1 (1:100), OATP4C1 (1:400), ABCB1 (1:400), ABCC3 (1:400), ABCC4 (1:400), and ABCC5 (1:400). Biotionylated secondary antibodies were detected using a streptavidin-bioptin-complex coupled to horse radish peroxidase and a diaminobenzidin stain, color-enhanced with CuSO_4_. Finally, the biopsies were periodic acid-Shiff-hematoxylin stained. For negative controls primary antibody was replaced by either normal goat (Dako X0902) or rabbit (Dako X0907) serum (1:100). Images were recorded using an Ortoplan microscope (Wetzlar, Germany) fitted with an Evolution MP camera (Silverspring, MD, USA) and analysis was performed using Image-Pro 5.0 software.

### Statistical analysis

Hypothesis testing was done by unpaired student’s *t*-test or Mann–Whitney rank sum test. P-values below 0.05 were considered significant. All SCC-signals obtained were included with the same weight regardless of the number of biopsies from each patient. This procedure was chosen in order to reduce the importance of outliers in the single patient. All statistics were performed using SigmaPlot 11.0 software.

## Results

### Functional characterization of transport

**Figure 1:**
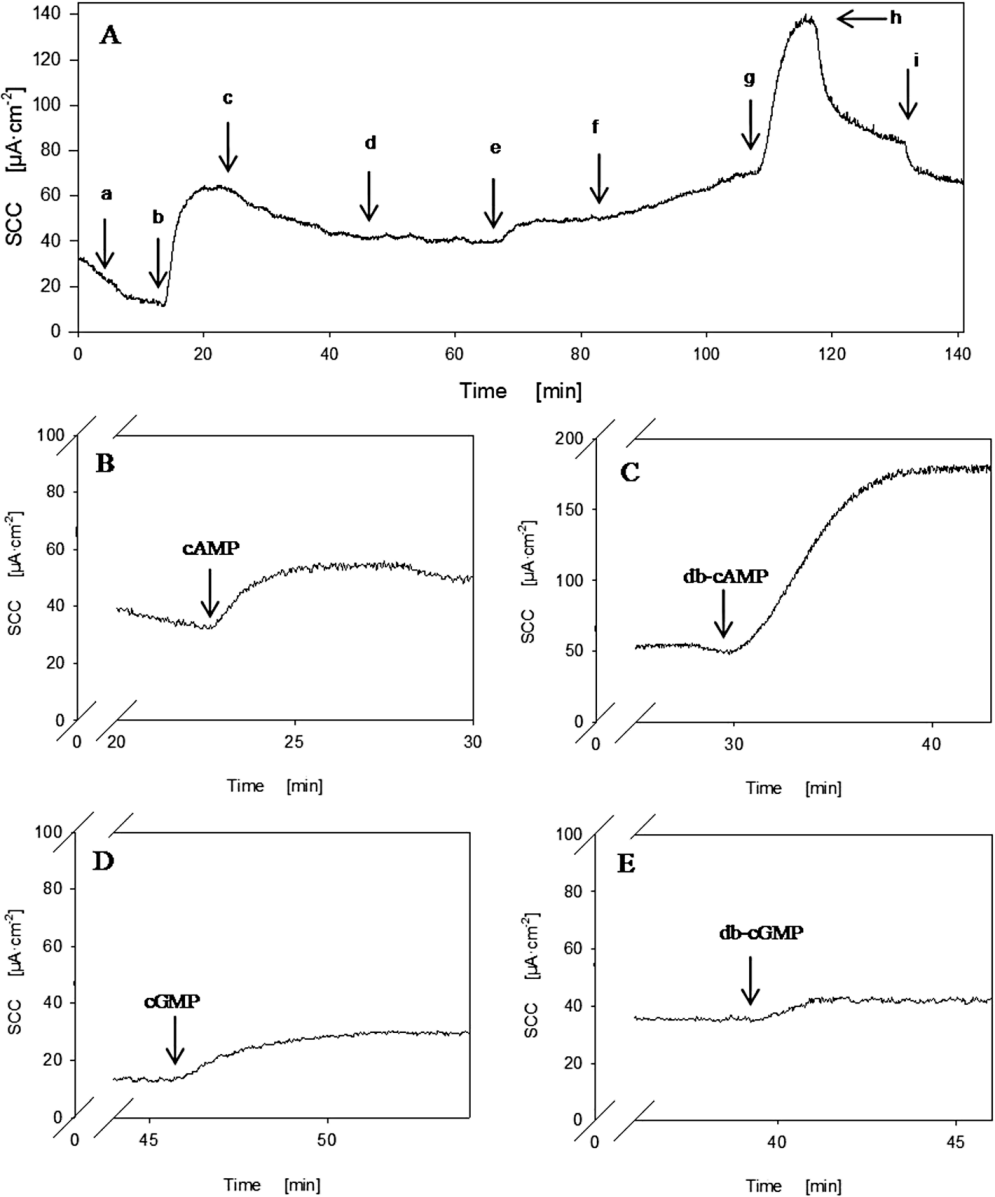
**Example of short circuit current data from a MUAS-chamber**. **A:** Typical responses from compounds applied apically and/or basolaterally. a: amiloride, b: theophylline, c: indomethacin, e: cAMP (basolateral), g: db-cAMP (basolateral), h: bumetanide, and i: acetazolamide. d: and f: apically applied cAMP and db-cAMP, respectively**. B-E:** Typical examples of induced SCC by the four model compounds.

**Figure 2:**
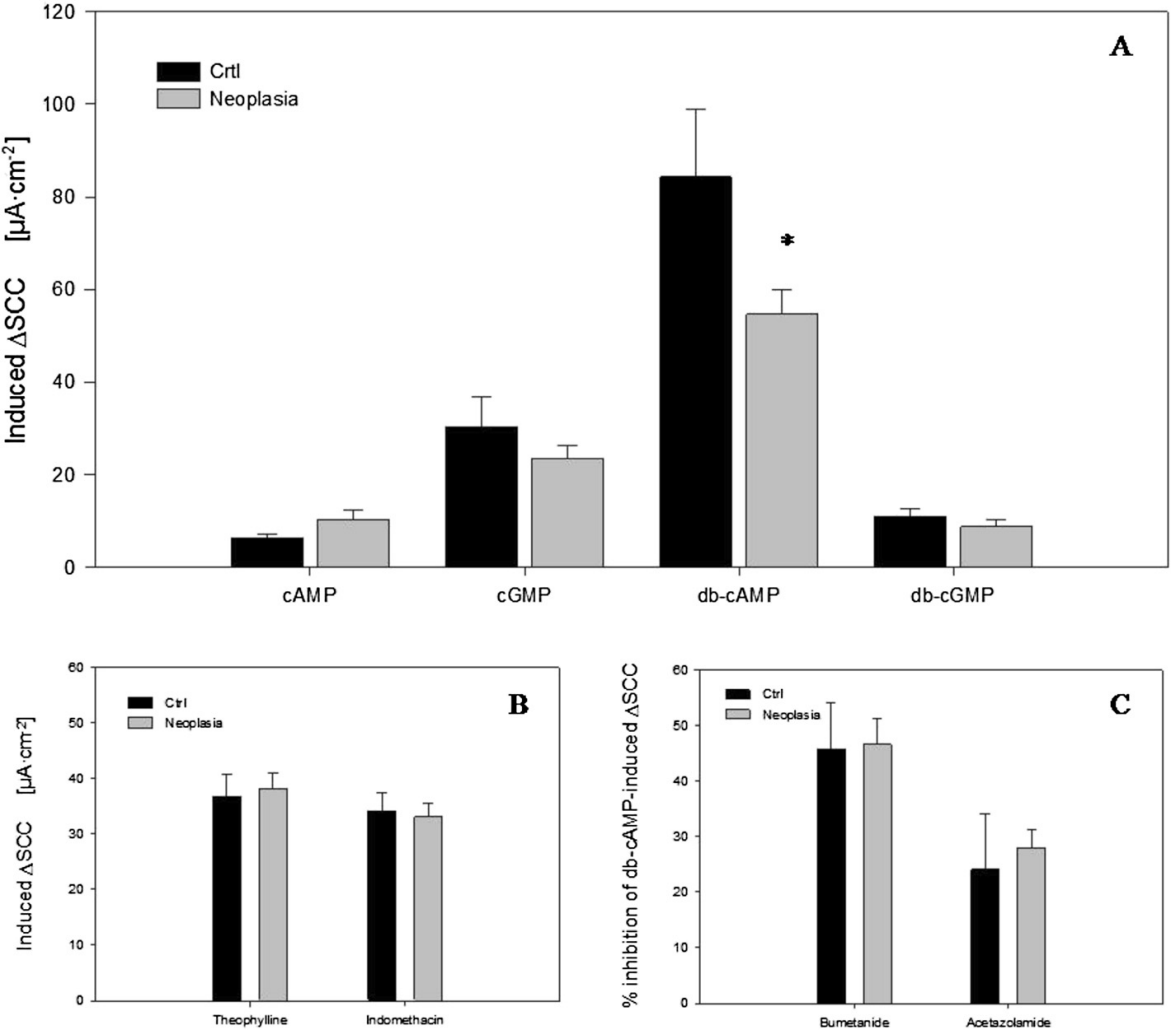
**Mean short circuit signals. A:** Induced SCC by 500 mM of the 4 cNTs presented as mean ± SEM. Control patients: cAMP (N = 7, n = 10), cGMP (N = 5, n = 7), db-cAMP (N = 7, n = 14), db-cGMP (N = 7, n = 12). Neoplasia patients: cAMP (N = 7, n = 12), cGMP (N = 10, n = 20), db-cAMP (N = 10, n = 32), db-cGMP (N = 4, n = 7). **B:** Responses to application of theophylline and indomethacin. Bars represent Mean ± SEM. Control patients: Theophylline (N = 8, n = 25), indomethacine (N = 8, n = 27). Neoplasia patients: Theophylline (N = 11, n = 37), indomethacine (N = 11, n = 41). **C:** Responses to application of bumetanide and acetazolamide. Bars represent Mean ± SEM. Control patients: Bumetanide (N = 6, n = 12), acetazolamide (N = 5, n = 11). Neoplasia patients: Bumetanide (N = 9, n = 24), acetazolamide (N = 9, n = 25). N = number of patients and n = number of biopsies. * indicates a statistical significant difference between the two groups.

From each patient three to four biopsies were successfully mounted. Slope conductance ranged between 30 and 60 mS·cm^-2^ during the observation period and with a stable baseline SCC (basal SCC). Examples of SCC responses to pretreatment and to cyclic nucleotide exposure are shown in Figure 1. When basolaterally applied, extracellular cAMP, cGMP, and db-cGMP gave rise to 10–20 μA·cm^-2^ secretory signals, implying a basolateral influx mechanism of cNTs into cells. The same was observed for basolateral db-cAMP, but this compound lead to a 5–10 fold larger response. Figures 1B-E show representative responses to serosally applied cNTs. The means of induced currents for all 4 compounds in each patient group are presented in Figure 2A. Contrary, apical addition of the four nucleotides had no effect on SCC. This is shown for cAMP and db-cAMP in Figure 1A, indicating db-cAMP as a non-permeable analogue in our experimental setting and implying a transport mechanism at the basolateral membrane.

As appears from Figure 2A, no significant differences in size of induced current were identified between patient groups for cAMP, cGMP or db-cGMP. However, for db-cAMP which induced the largest signal, the increase in SCC was significantly lower in biopsies from patients with neoplasia (p = 0.024). The lower response to transported db-cAMP in CRN patients could be due to altered transport function and/or altered expression or activity of responsible transporters. Alternatively, a mechanism downstream in the cAMP-activated pathway might be altered, see Discussion.

The induced SCC-signals were subsequently to a large extent inhibitable with bumetanide (Figure 1A) implying that mainly a Cl^-^-secretion was responsible for the cNT-induced increase in SCC. Addition of acetazolamide further decreased the SCC (Figure 1A), indicating that intracellular bicarbonate was contributing to the observed secretory response. The inhibitory effects of bumetanide and acetazolamide are summarized in Figure 2C.

Induced SCC unaffected by bumetanide and acetazolamide accounted for up to 25% and in order to identify the mechanisms behind this current, inhibitors of co-transporters AE1/2 and NBC were applied separately. No effect was observed with addition of SITS, but NPPB caused a large decrease in SCC, resulting in a complete inhibition of the remaining db-cAMP-induced current (data not shown). This suggests that a sodium coupled bicarbonate transport of exogeneous bicarbonate available in the Ringer solution is probably also responsible for the least 25% of the cNT-induced SCC.

### Differential transporter expression in biopsies

**Figure 3:**
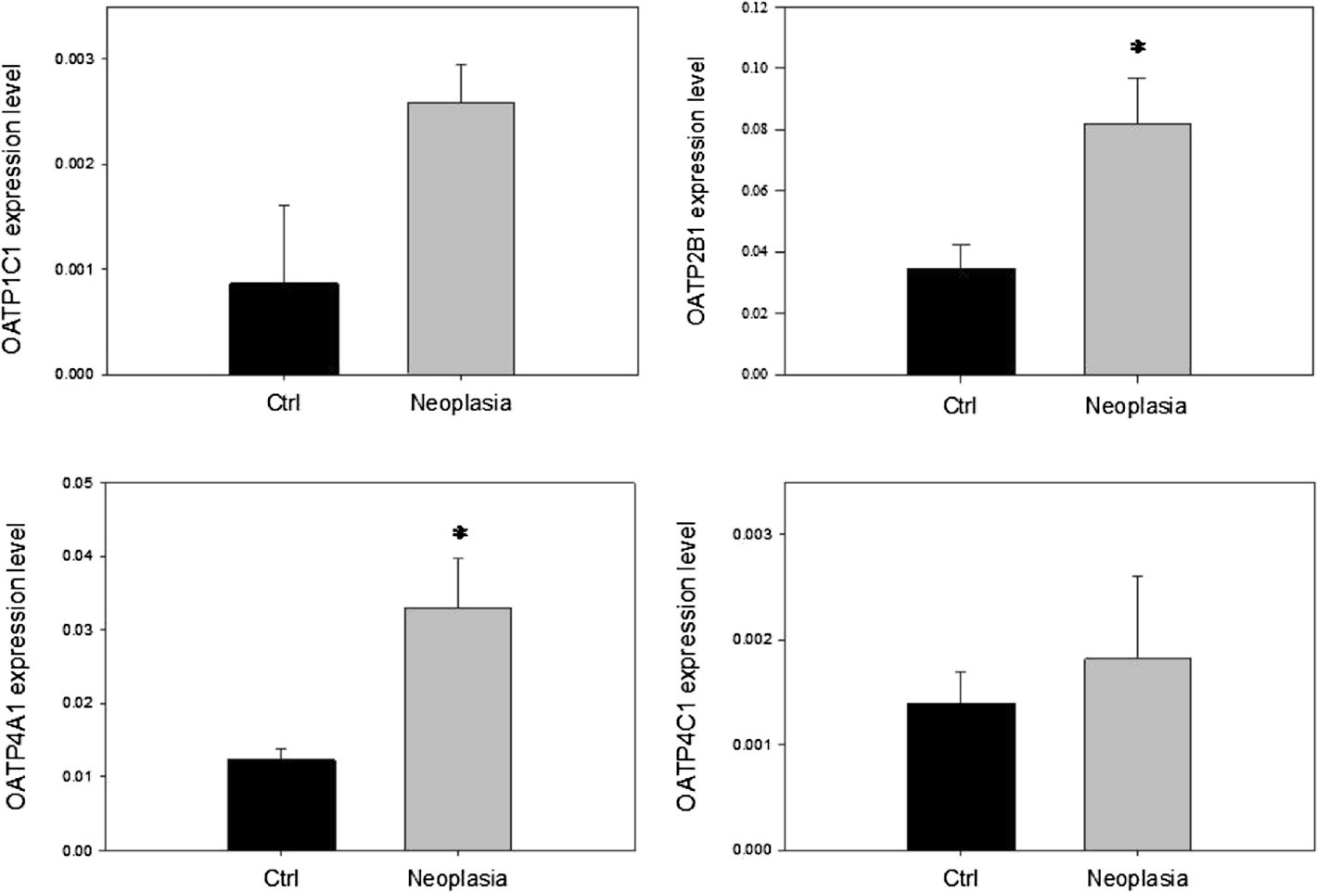
**OATP expression.** Expression levels of the OATPs relative to β-actin based on single biopsies from 6 patients in each group. * indicates a statistical significant difference (p < 0.05) between the two groups. Data are presented as means ± SEM.

All eight included transporters were detected in biopsies by rt-qPCR. Relative expressions of OATP- transporters are presented in Figure 3. Only OATP2B1 and OATP4A1 showed significantly altered expressions in patients with neoplasia compared to controls. Both were expressed significantly higher in biopsies from neoplasia patients (p = 0.017 and 0.013, respectively).

**Figure 4:**
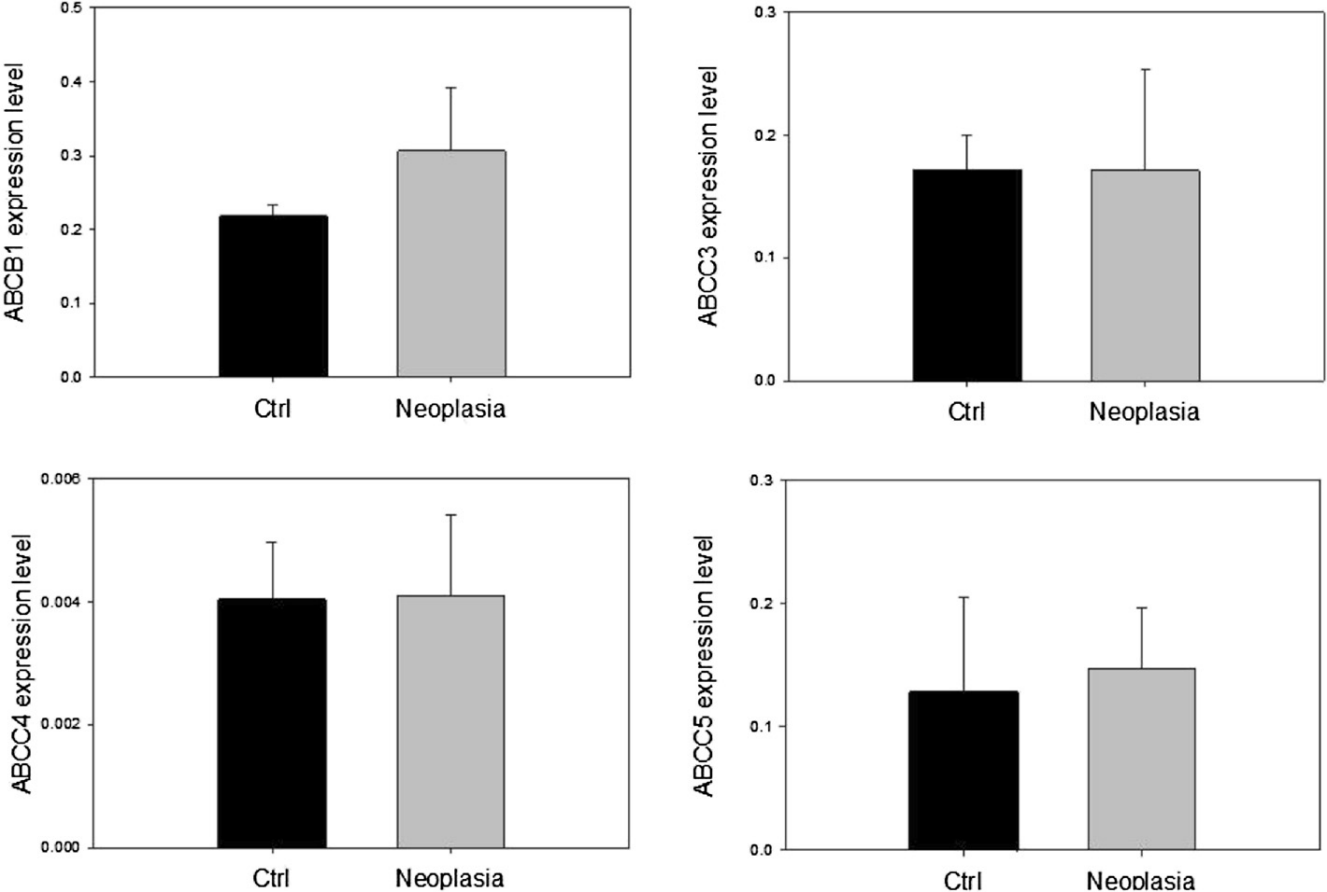
**ABC expression.** Expression levels of the ABC-transporters relative to β-actin based on single biopsies from 6 patients in each group. Data are presented as means ± SEM.

Relative expressions of ABC-transporters are shown in Figure 4. ABCB1 had the highest expression level. ABCC3 and ABCC5 were expressed to nearly the same extent, whereas ABCC4 was sparsely expressed compared to the other ABC-transporters. None of the transporters positively demonstrated to transport cAMP and cGMP [11,12,22] were over- or under-expressed between patient groups as could be expected from functional studies. This could imply an altered activity in transporters of both the OATP- and ABC-families, rather than expression being responsible for the functional difference observed in MUAS chamber studies, Figure 2.

### Transporter localization

**Figure 5:**
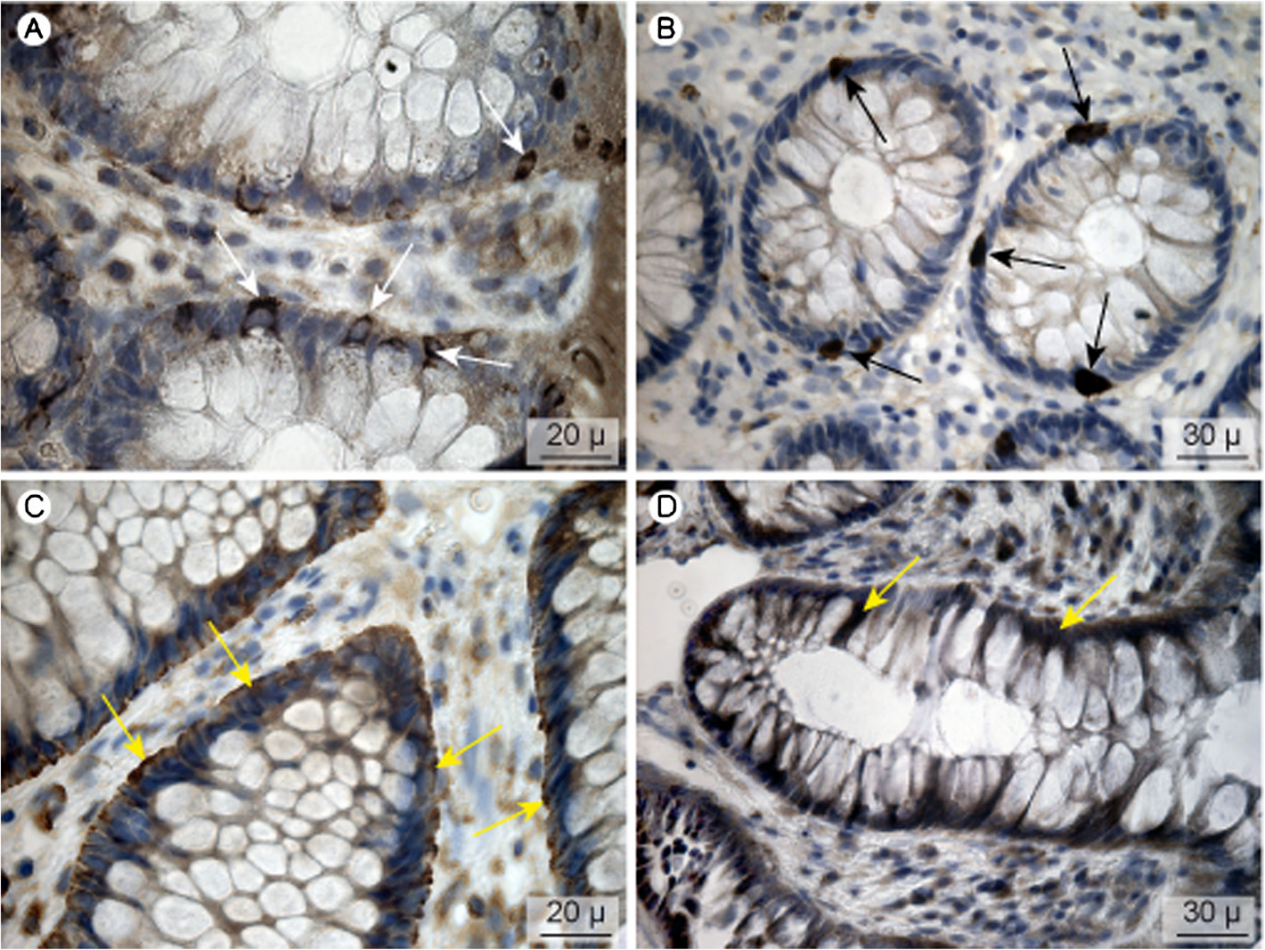
**OATP-transporter localization.** A: OATP1C1, B: OATP2B1, C: OATP4A1, D: OATP4C1. White arrows indicate the location at the bottom of the goblet cells, black arrows staining of endocrine cells and yellow arrows basolateral staining of epithelial cells.

Figure 5 shows images of biopsy specimens treated with OATP antibodies. OATP1C1 was detected at the basolateral membrane of goblet cells, whereas no staining was observed in columnar epithelial cells, Figure 5A. The OATP1C1 transporter is a known hormone and xenobiotics transporter with high affinity for tyroxine [16]. Its basal location in goblet cells therefore indicates a secretory function here for hormones and xenobiotics. OATP2B1 was exclusively detected in endocrine cells dispersed between columnar epithelial cells and goblet cells, Figure 5B. This indicates a regulator function rather than a direct excretory function. OATP4A1 was detected at the basolateral membrane of columnar epithelial cells, indicating a putative excretory function of this transporter, Figure 5C.

The immuno-reaction for OATP4C1, although weak, was located to the basolateral aspect of goblet and columnar epithelial cells, Figure 5D. This localization is similar to the position in the kidney tubule. As this transporter carries cNTs, it may account for a low capacity inward flux of externally applied cNTs as measured indirectly in this study, Figures 1 and 2[11].

**Figure 6:**
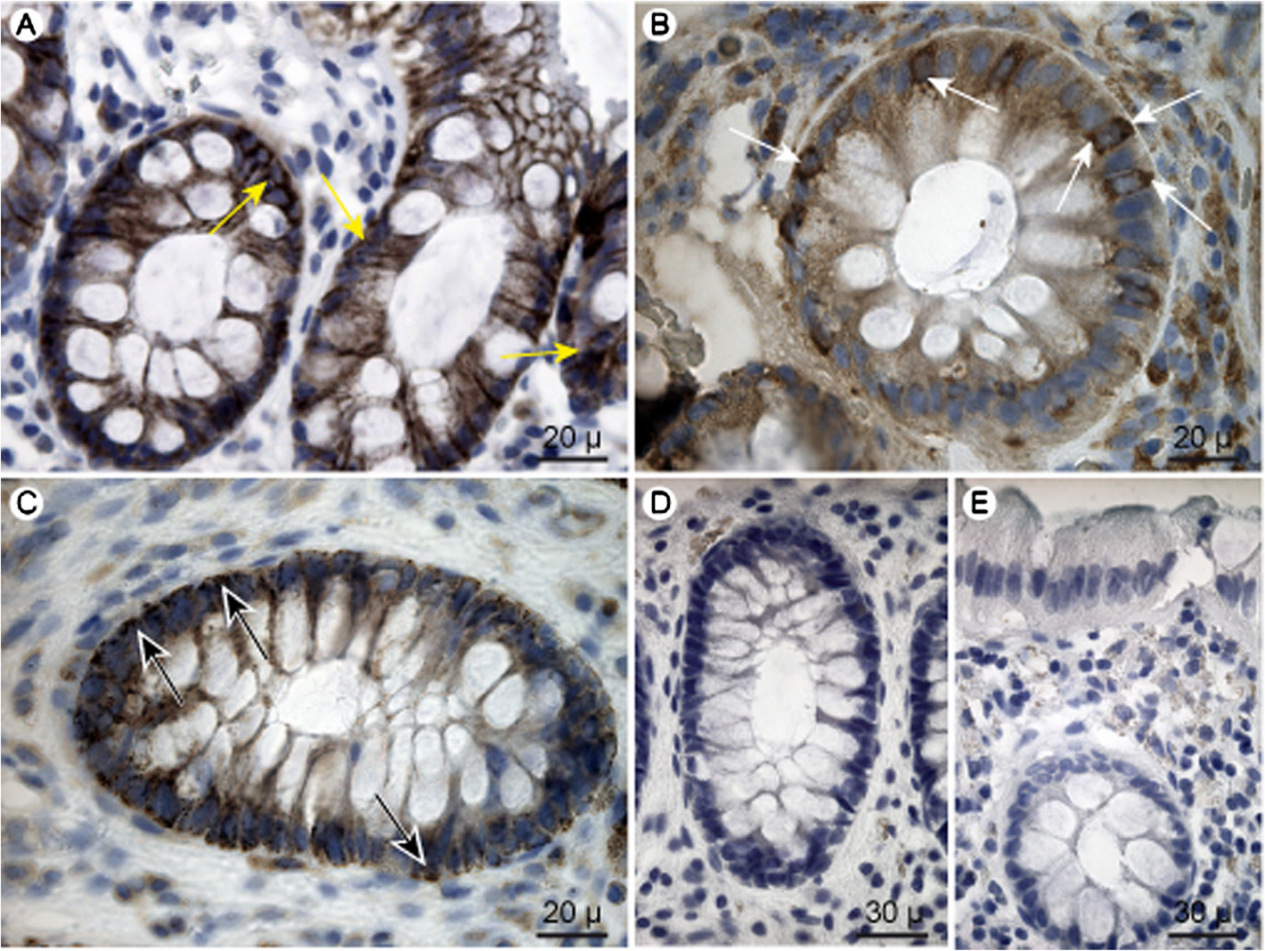
**ABC-transporter localization.** A: ABCC3, B: ABCC4, and C: ABCC5. Yellow arrows indicate the basolateral staining of ABCC3 in coloumnar epithelial cells and goblet cells. White arrows indicate the localization of ABCC4 in basolateral and perinuclear membranes of goblet cells, and black arrows indicate the basolateral staining of ABCC5 in columnar epithelial cells. D-E: Negative control images of staining with secondary antibody from goat (D) and rabbit (E). No immunohistochemical coloring was obtained for the ABCB1 transporter.

The subcellular localizations of the ABC-transporters are presented in Figure 6. ABCC3 was found to be located basolaterally in both columnar epithelial cells and goblet cells. This is in accordance with other studies [23]. Interestingly, ABCC4 was not detected in the plasma membrane of epithelial cells, but in the basolateral aspect and perinuclear membranes of goblet cells, Figure 6B. In other studies it has been found in the apical membrane of tubular cells in the kidney and basolateral membrane of the liver and small intestine [23]. ABCC5 was located at the basolateral membrane of columnar epithelial cells suggesting that this transporter very well could be implicated in local excretion of xenobiotics as well as occupying a role in intracellular levels of cAMP and cGMP. Localization of ABCB1 could not be determined, as the chosen antibody did not complex with possible epitopes (4 experiments, data not shown), but its localization is well described and confined to the apical membrane of human colonic epithelial cells [24,25].

No differences in localization between the two groups of patients were observed in biopsies subjected to immunohistochemistry.

### Studies with rifamycin

Rifamycin SV was added in an attempt to inhibit the effects of db-cAMP, as rifamycin SV is known to inhibit several of the OATP transporters with an IC_50_ around 1 μM [18,19]. Meanwhile, rifamycin had no effect on basal or db-cAMP-induced SCC, data not shown. The employed concentration of rifamycin gave a clear yellow coloring of the media, and since the coloring was not transferred to the other half chamber during at least two hours of experimentation, we conclude that the media on either side of the tissue are fairly well separated.

### Tissue integrity

A total of 76 biopsies from 19 patients were included in the study and the overall structure of the biopsies was well kept, as approximately 80% were intact or only slightly damaged. Scoring of biopsies after end of experiment in MUAS-chambers was as follows: 0 (17), 1 (45), 2 (9), 3 (3). Data from biopsies assigned to category 3 were excluded from the statistics. Two biopsies were pinched deeper and the surface epithelium could not be assessed. However, the signals achieved from these biopsies fell within the range of the data and they were included in the statistics.

## Discussion

The present study was initiated to further elucidate two aspects of the normal function of human colon epithelium, *viz*., colon’s epithelial excretory system of toxic and waste molecules and colon’s use and turnover of second messenger cAMP. Additionally, we have questioned if these functions are altered in the neoplastic condition of the colonic tissue.

### The excretory system of human colon epithelium

**Figure 7:**
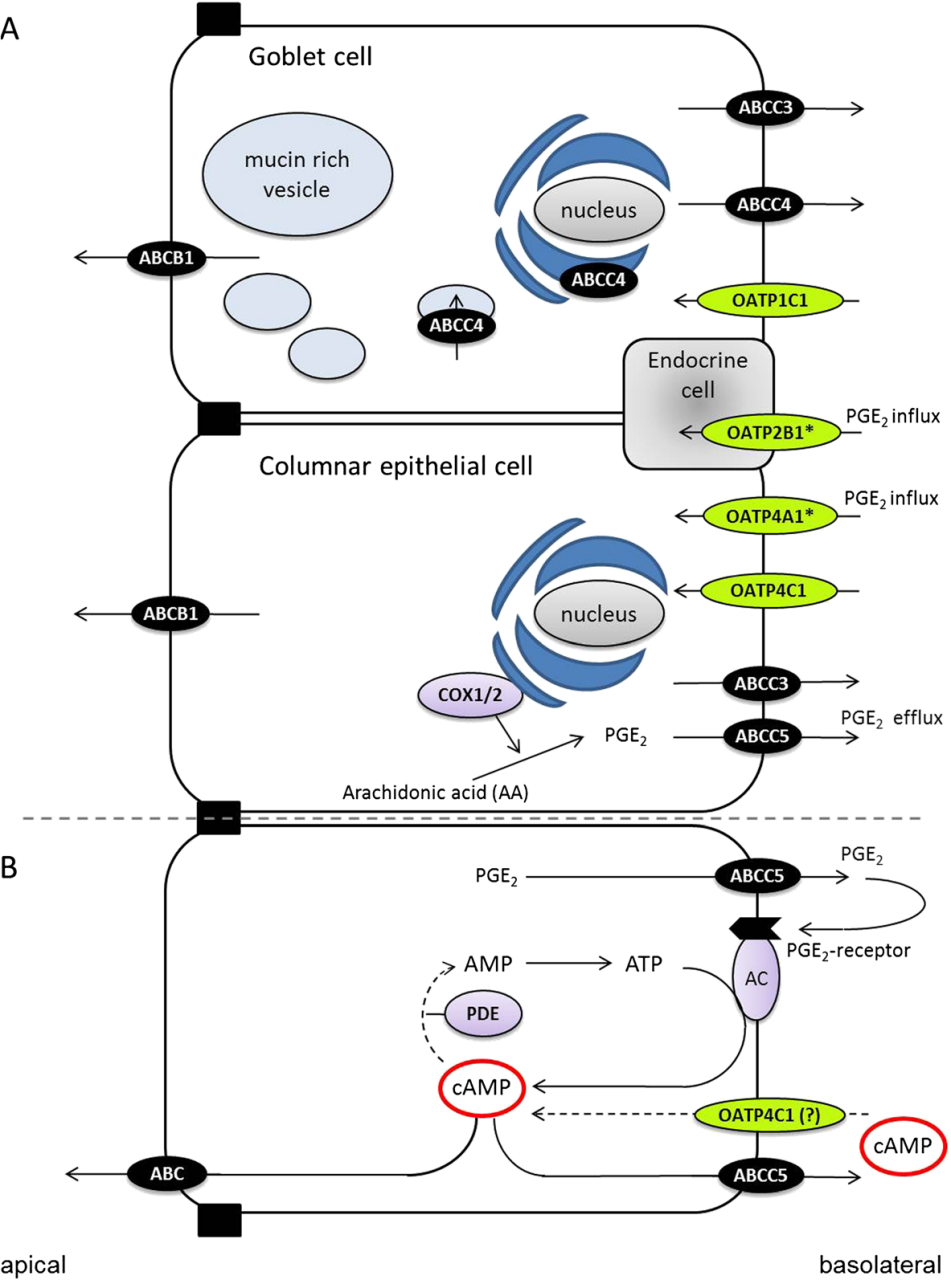
**Model of human colon secretory and cAMP turnover systems.** A: Schematic presentation of the secretory system of the colon as revealed by this study. The subcellular location of ABCB1 was not determined in this study but has been localized to the apical membrane by others. * Indicates significantly increased expression in CRN patients. B: Schematic presentation of the cAMP-turnover system in a colonic epithelial cell. AC: adenylate cyclase. PDE: phosphodiesterase.

Recently, more detailed understanding has emerged of how the body rids itself of waste metabolites and toxic products. Indeed, three epithelia are in focus: cells of the bile canaliculus, the kidney tubule, and the colon mucosa [13,15,23,26]. Of these three epithelia, the colonic excretory system is the least studied, in spite it is involved in the final steps for excretory processes of the gastrointestinal tract [14,26]. We therefore decided to characterize the expression and localization of selected ABC- and OATP transporters assumed to be involved in the excretory mechanisms as well as in the handling of cAMP in human colon. The analysis of expression and localization of transporters are shown in Figures 3,4,5 and 6 and a summary of the results with a model of the colonic excretory system is shown in Figure 7A. The localization of transporters needs affirmation, e.g. by a double labeling technique. For the regulated turnover of cAMP in human colon, a model is presented in Figure 7B.

#### Probing the excretory system with cNTs

We have studied effects of externally applied cyclic nucleotides, cAMP, cGMP, db-cAMP and db-cGMP, on induction of some aspects of the excretory process in biopsies of human colon mucosa. The results are shown in Figures 1 and 2 and indicate that cNTs are carried by specific transporters across the basolateral membrane of epithelial cells, while the luminal cell membrane lacks these transporters as no anion secretory response can be induced when applying the cNTs in the luminal media, Figure 1A.

Since the blood side concentration of cNTs, used to obtain a sizable response was rather high (sub-millimolar range), compared to the cNT concentration (around 1 μM) known to be effective when present in the cytoplasm [27], this indicates that the transport of cNTs is of a limited capacity as the induction of a secretory response is a balance between influx and elimination of cAMP. The elimination of intracellular cAMP is effectuated by residual phosphodiesterase activity and active export, see below. A likely candidate for the influx transport of cNTs is the influx digoxin transporter OATP4C1 known to also transport cNTs [11] and regarded as a kidney specific transporter implicated in the removal of uremic toxins, waste products, and drugs [28]. The necessary high cNT concentration needed to elicit an effect also seems to exclude an interaction with a possible cNT receptor on colonic epithelial cells similar to the cAMP induction of spore formation for the slime mould D*ictoystelium discoideum* at an external cAMP receptor with an EC_50_ around 1 μM [29].

Moreover, when applied externally, db-cAMP is several fold more efficacious in inducing a secretory response than the other three employed cNTs, including cAMP, as can be seen from the results in Figure 2A. One likely explanation for this observation is that a basolateral influx transporter is more selective for db-cAMP with higher affinity and/or efficacy. Other possible explanations are that the sensitivity of cytoplasmic cAMP receptors, such as the regulatory unit of protein kinase A, is higher to db-cAMP than to cAMP itself or alternatively that the breakdown to non-cyclic nucleotide of cyclic nucloetides by phophodiesterases in the cytoplasm is less efficient for db-cAMP than for the other cNTs; as demonstrated several years ago in cell cultures [30]. Yet, another possibility is a less efficient active export of db-cAMP by ABCC5 as compared to for instance cAMP, see next section.

It may be noted, that for isolated hen colon compared to human colon, the ratio effect on SCC by cAMP and db-cAMP is reversed. By similar external additions to the basolateral side of hen colon, cAMP increases the SCC (134 +/− 11 μA,cm^-2^, n = 74) 6 fold above that for db-cAMP (Bindslev, unpublished data).

#### Model for colonic handling of cAMP and other cyclic nucleotides

Based on results obtained in this study with expression and localization of transporters in the human colonic mucosa, Figures 3,4,5 and 6, and other evidence in the literature [22], we propose a model for and give a discussion of the handling of cAMP in human colonic epithelium as shown in Figure 7B in relation to production, compartmentalization, degradation and active export of this second messenger; and possible relevance to development of colorectal cancer [17,31].

One of the regulatory pathways for synthesis of cAMP is the step starting with COX enzymes producing prostaglandins of the E series (e.g., PGE_2_) which activate EP subtype specific receptors (especially the EP4 subtype receptor in human colon) [17], transducing the signal through Gαs proteins to various isoforms of the adenylate cyclase enzyme, AC [32,33]. cAMP is confined to a submembraneous compartment just under the basolateral membrane from where it is eliminated by either of two pathways [12,34]. One down-regulatory pathway is the classical degradation of cAMP by cAMP-specific phosphodiesterases, PDEs. For the anion secretory process in human colon epithelium, the PDEIII subtype seems to be most important (unpublished data by dr. G. Majgaard). db-cAMP is known to be more resistant to PDE degradation in cell systems than cAMP and this could explain its observed higher efficacy [30]. Another possible explanation for the observed difference in effect of db-cAMP in the two patient groups could hence be due to an increase in PDE activity or lowered sensitivity of PKA to db-cAMP in the CRN group.

Another well-described down-regulatory pathway for cAMP is via export pumps at the basolateral membrane from the nearby cAMP compartment, Figure 7B [34,35]. The most likely export pump for cAMP in the human colon epithelium is the ABCC5 transporter as it is localized to the basolateral cell membrane, Figure 6C. Usually the ABCC4 transporter is considered the most potent cAMP export pump among the known cAMP exporters, ABCC4, ABCC5, and ABCC8 [22]. However, the ABCC4 transporters do not seem to be located in the basolateral cell membrane of epithelial columnar cells but rather in basolateral membrane as well as perinuclear and secretory vesicle membranes of goblet cells, Figure 6B. The function of this export pump here is unknown so far, but may be involved in an export of antimicrobial proteins similar to defensins [36] coming from colon crypts cells, CD24(+); residing between Lgr5 stem cells representing Paneth cell equivalents in colon [37].

Influx of cNTs across the epithelial basal membrane can explain the observed induction of ion secretion by externally applied cNTs. cNT influx we ascribed to OATP4C1, which is a known cAMP influxer [11] and expressed in human colonic tissue, Figure 3D [14], at the basolateral membrane of colonic epithelial and goblet cells, although only weakly visible, Figure 5 D. Thus, a firm proof of OATP4C1’s localization and function must await further studies.

#### Alterations with neoplasia of cNT transport and transporter expression in the colonic excretory system

As shown in Figure 2, the induction of a colonic secretory response by db-cAMP was significantly lower in CRN affected patients compared to non-CRN patients. None of the studied colonic transporters known to be involved in influx and/or efflux of cNTs, i.e., OATP4C1, ABCC4 and ABCC5, were significantly altered between CRN and non-CRN patients, Figures 3 and 4. This points to a lower sensitivity to db-cAMP in the PKA pathway of colonic mucosa from CRN patients, rather than an altered membrane transport rate between the two patient groups, e.g., a reduced influx or an augmented efflux rate for the CRN colon, Figure 7B.

#### Expression and location of other transporters in relation to CRN

The significant increase in PGE_2_- influx transporters OATP2B1 and OATP4A1 in colon biopsies from CRN-patients, Figure 3, may indicate that these transporters are somehow involved in a mediated excretory function, up-regulated with neoplasia. However, our negative results with rifamycin which is a rather potent inhibitor of these two OATP-transporters seem to indicate that neither OATP2B1 nor OATP4A1 are involved in the basolateral cNT transport. Furthermore, if OATP4A1 transported cNTs into colonic mucosal cells, this does not fit with its increased expression in non-CRN and a lower transfer in colon from CRN affected patients.

#### COX enzymes, NSAID and PGE_2_ transporters in human colon

It has been known since the 1960s that drugs of the NSAID-type in doses as low as 10–30 μM can ameliorate the course of colorectal cancer [31,38]. In view of NSAIDs as classical inhibitors of COX enzymes, their effect is also to lower production of prostaglandins, in human colon, as for instance PGE_2_ and PGF_2_-alfa and thereby the content of second messengers as cAMP and other cyclic nucleotides, cNTs, in epithelial cells [39].

The export and import of PGE_2_ in normal, native colonic mucosa has so far been assumed via transporters as ABCC4 and OATP2A1 (prostaglandin transporters) [12], presumably also in the basolateral epithelial cell membrane. The basolateral PGE_2_-export should maybe rather be ascribed to ABCC5 as found basolaterally in this study and the import also possibly related to a new PGE_2_ OAT-transporter found in colonic tissue [40]. Furthermore, OATP2B1 and OATP4A1 are both up-regulated in CRN-patients and known to transport PGE_2_[15,16,41]. Therefore, studies combining effects of all PGE_2_ transporters, their expression and localization in normal human colonic mucosa are warranted.

## Conclusions

The SCC induced by db-cAMP in CRN-patients is significantly lower than in non-CRN patients. This implies a correlation between CRN and lower transport of or sensitivity to db-cAMP. The study has ascertained ABCC5 and excluded ABCC4 as a cNT efflux pump in basolateral membranes of human colonic epithelial cells. ABCC4 is rather located to the basolateral and perinuclear membranes of goblet cells. OATP4C1 is a likely influx transporter of cNTs in human colon epithelial cells, but this needs additional investigations to be confirmed. Furthermore, there is a higher expression of OATP4A1 and OATP2B1 (both influxers of PGE_2_) in colon from CRN-patient compared to non-CRN-patients. How this altered expression correlates with an observed lower secretion induced by db-cAMP in CRN is unresolved, but may indicate a CRN-induced removal of known augmented colonic content of prostaglandins.

## Abbreviations

ABC, ATP-binding cassette; cNTs, cyclic nucleotides; CRC, Colorectal cancer; CRN, Colorectal neoplasia; db-cAMP, Dibutyryl-cyclic adenosine monophosphate; db-cGMP, Dibutyryl-cyclic guanosine monophosphate; IC50, Half maximal inhibitory concentration; MUAS, Mini-Ussing air-suction; NPPB, 5-nitro-2-(3-phenyl-propylamino)-benzoic acid; OATP, Organic anion transporting polypeptide; SITS, 4-acetamido-4'-isothiocyano-2,2'-disulfonic stilbene; SCC, Short circuit current.

## Competing interests

The authors declare no conflicts of interest

## Author’s contributions

KK was responsible for and conducted all experiments, data analyses and writing of the manuscript. GMJ was involved in the functional studies. ML, DP, and LGG were involved in the experimental design and interpretation of the rt-qPCR studies. SSP was involved in the planning and interpretation of immunohistochemical studies. SK was responsible for the endoscopy procedures. MBH and NB conceived of the study, supervised the experimental work, and were involved in the experimental design and writing of the manuscript. All authors read and approved the final manuscript.

## Pre-publication history

The pre-publication history for this paper can be accessed here:

http://www.biomedcentral.com/1471-230X/12/78/prepub
